# High Prevalence of Vitamin D Insufficiency in China: Relationship with the Levels of Parathyroid Hormone and Markers of Bone Turnover

**DOI:** 10.1371/journal.pone.0047264

**Published:** 2012-11-08

**Authors:** Han-Kui Lu, Zeng Zhang, Yao-Hua Ke, Jin-Wei He, Wen-Zhen Fu, Chang-Qing Zhang, Zhen-Lin Zhang

**Affiliations:** 1 Department of Nuclear Medicine, Shanghai Jiao Tong University Affiliated Sixth People's Hospital, Shanghai, People's Republic of China; 2 Metabolic Bone Disease and Genetic Research Unit, Department of Osteoporosis and Bone Diseases, Shanghai Jiao Tong University Affiliated Sixth People's Hospital, Shanghai, People's Republic of China; 3 Department of Orthopedic Surgery, Shanghai Jiao Tong University Affiliated Sixth People's Hospital, Shanghai, People's Republic of China; Virginia Commenwealth University, United States of America

## Abstract

There is a lack of large-scale studies on vitamin D status and its relationship to parathyroid hormone (PTH) and bone turnover markers in adults living in Shanghai. The objectives were to determine the prevalence of vitamin D insufficiency in Shanghai and to investigate the relationship of 25(OH)D with parathyroid function and bone turnover markers. This cross-sectional study involved 649 men and 1939 women aged 20–89 years who were randomly sampled in Shanghai. Serum concentrations of 25(OH)D, PTH, albumin, and bone turnover markers were measured. During the winter season, the prevalence of vitamin D insufficiency (<30 ng/mL) was 84% in males and 89% in females. The prevalence of vitamin D deficiency (<20 ng/mL) was 30% in males and 46% in females. With increasing serum 25(OH)D concentrations categorized as <10, 10–20, 20–30, and ≥30 ng/mL, the mean PTH and bone turnover markers levels gradually decreasd in both sexes (p<0.001). There was an inverse relationship between the serum 25(OH)D and PTH concentrations in both genders, but no threshold of 25(OH)D at which PTH levels plateaued was observed. There were modest but significantly inverse relationships between the levels of 25(OH)D and bone turnover markers, but no plateau was observed for serum 25(OH)D levels up to 40 ng/mL.

## Introduction

Vitamin D is known to be an essential element for bone metabolism and skeletal health. In recent years there has been increasing interest in the influence of vitamin D on extraskeletal health. Indeed, vitamin D status may play a role in diabetes mellitus [1], [2], cancers [3], multiple sclerosis [4] and other autoimmune diseases [5], [6], cardiovascular diseases [7], and infectious diseases [8].

The major source of vitamin D for most humans is exposure to sunlight. Vitamin D3 is synthesized in human skin via the photoisomerization of 7-dehydrocholesterol (7DHC) to yield previtamin D3 during exposure to UVB radiation [9]. Previtamin D3 then undergoes hydroxylation in the liver, resulting in the formation of 25-hydroxyvitamin D3 [25(OH)D3], the primary circulatory form. 25(OH)D3 subsequently undergoes hydroxylation in the kidney to yield the biologically active form of vitamin D, calcitriol [1,25-(OH)2D]. For adults in a bathing suit, exposure to the amount of sunlight that causes a slight pinkness of the skin 24 hours later (1MED) is equivalent to ingesting approximately 20,000 IU of vitamin D [6]. Very few foods naturally contain vitamin D, and foods that are fortified with vitamin D are often inadequate to satisfy either a child's or an adult's vitamin D requirement [10].

The serum level of 25(OH)D is a reliable indicator of the vitamin D status of an individual [11]. Although vitamin D deficiency has been defined, there is no consensus on a cut-off level for defining vitamin D deficiency. Nevertheless, it is widely suggested that the circulating 25(OH)D concentrations of less than 30 ng/mL should be considered indicative of vitamin D insufficiency, whereas circulating concentrations of less than 20 ng/mL should be considered indicative vitamin D deficiency [12]. Using these definitions, it has been estimated that between 40% and 100% of elderly US and European non-hospitalized men and women are either vitamin D deficient or insufficient [13]. High rates of vitamin D deficiency have been reported in children and adults all over the world [14]. Recently, Wat et al. [15] have shown that southern Chinese adults exhibit high prevalence (62.8%) of vitamin D insuffficiency [25(OH)D<30 ng/mL] in association with secondary hyperparathyroidism. Zhao et al. [16] showed 25(OH)D was negatively correlated with beta C-terminal cross-linked telopeptides of type I collagen (β-CTX) and amino-terminal propeptide of type I collagen (P1NP) in Chinese postmenopausal women from Beijing. China covers a large area and regions at different latitudes receive different amounts of sunlight. Shanghai, located on the eastern coast of China with a subtropical latitude of 31°N, is a flourishing international metropolis with a population of over 30 million. There is a lack of large-scale studies on vitamin D status and its relationship to parathyroid hormone (PTH) and bone turnover markers in adults living in Shanghai. Therefore, the objectives of our study were to determine the prevalence of vitamin D insufficiency in Shanghai, and to investigate the relationship between 25(OH)D and both parathyroid function and bone turnover markers.

## Methods

### Study population

From February 2009 to March 2009, 2243 women (aged 20–96 years) and 865 men (aged 23–94 years) - a total of 3,108 healthy Chinese people living in Shanghai - were recruited from several community centers. The study subjects were identical to those included in the Shanghai Osteoporosis Study (SOS). The present study assessed the physical health of the participants, including heart, liver, kidney, and bone health. The study subjects were randomly recruited from among the inhabitants of ten communities of Shanghai according to population composition. We included persons who were confirmed to be living in Shanghai for at least 5 years and listed in the residential registration record. After stratifying the population of each selected community by age, we randomly sampled subjects. Selected participants were called and persuaded to participate in this study. All subjects who agreed to participate in this study were asked to visit an outpatient clinic at the Department of Osteoporosis and Bone Diseases, Shanghai Jiao Tong University Affiliated Sixth People's Hospital. All participants were of Han ethnicity. Age, body weight, height, and age of menarche and amenorrhea were recorded. All subjects were medically examined and interviewed using a standardized questionnaire to collect information on life style, smoking habits, the level of physical activity during leisure time, and use of vitamins and medications. Dietary calcium intake was calculated using a three food questionnaire.

All subjects were subjected to blood counts, fasting plasma glucose tests, liver and kidney function tests. All healthy subjects included in the present study had (1) normal blood counts and (2) normal results for liver and kidney function tests. In addition, no participant was receiving treatment or had medical complications known to affect bone metabolism, including cancer, hyperthyroidism, diabetes mellitus, primary hyperparathyroidism, pituitary, adrenal and rheumatic diseases. Participants who had taken vitamin D and/or calcium supplements within 3 months were also excluded.

The study was approved by the Ethics Committee of the Shanghai Jiao Tong University Affiliated Sixth People's Hospital. All participants signed informed consent forms before entering the study.

### Blood collection and analysis

Fasting blood samples were collected for the measurement of the serum levels of calcium, phosphate, albumin, glucose, insulin, cholesterol, triglycerides (TG), blood urea nitrogen (BUN), creatinine (Cr), alanine aminotransferase (ALT), aspartate transaminase (AST), r-glutamyl transpeptidase (r-GT), alkaline phosphatase (ALP), intact parathyroid hormone (iPTH), beta C-terminal cross-linked telopeptides of type I collagen (β-CTX), amino-terminal propeptide of type I collagen (P1NP), osteocalcium (OC) and 25-hydroxyvitamin D [25(OH)D]. Serum levels of 25(OH)D, PTH, β-CTX, intact-OC, and P1NP were determined using an ECLIA Elecsys autoanalyzer (E170; Roche Diagnostic GmbH, Mannheim, Germany). The intra-assay coefficients of variation (CVs) for 25(OH)D were 5.7% at a level of 25.2 ng/mL, 5.7% at a level of 39.9 ng/mL and 5.4% at a level of 65.6 ng/mL, respectively. The inter-assay CVs for 25(OH)D were 9.9% at a level of 25.2 ng/mL, 7.3% at a level of 39.9 ng/mL and 6.9% at a level of 65.6 ng/mL, respectively. The lower limit of detection of 25(OH)D was <4 ng/mL (10 mmol/L). The intra-assay and inter-assay CVs were 1.4% and 2.9%, respectively, for PTH, 2.5% and 3.5%, respectively, for β-CTX, 2.9% and 4.0%, respectively, for OC, and 2.3% and 2.8%, respectively, for P1NP. All serum samples were taken during the winter season (from February 2009 to March 2009).

### Statistical analysis

Continuous variables are presented as means (±SD). Data that are not normally distributed are shown as medians and inter-quartile ranges (in parentheses). Results that were not normally distributed were log transformed before analysis. Student's t-test was used to compare the mean values of continuous variables. Because of sex interactions, we analyzed the data separately for men and women. Circulating 25(OH)D concentrations were divided into four subgroups according to the following criteria: severely deficient (<10 ng/mL), deficient (≥10 and <20 ng/mL), insufficient (≥20 and <30 ng/mL), and sufficient (≥30 ng/mL). ANOVA was used to assess differences in age, BMI and the levels of serum PTH, bone turnover markers, and albumin among different 25(OH)D subgroups. Pairwise comparisons for multiple comparisons (Bonferroni) were also performed if the one-way ANOVA was significant. Locally weighted regression smoothing (LOESS) plots were performed to study the correlation of 25(OH)D with PTH and bone turnover markers. Potential confounders included age and BMI. First, unadjusted analyses were performed. Subsequently, potential confounders were added to the methods. All calculations were performed using SPSS ver. 11.0 (SPSS Inc., Chicago, IL, USA). Results were regarded as statistically significant at a value of p<0.05.

## Results

Based on the results of the biochemical tests, physical examinations and detailed questionnaires, we excluded 404 females and 216 males. Finally, 1839 women and 649 men were included. The clinical characteristics and mean laboratory values of the 1839 women and 649 men, aged 20–89 years, are shown in Table 1. All participants were of Han ethnicity and residents of Shanghai. The overall median (inter-quartile range) serum concentrations of 25(OH)D, PTH, β-CTX, OC and P1NP were 20.9 (16.9–25.0) ng/mL, 33.7 (26.3–43.6) pg/mL, 320.0 (220.0–460.0) pg/mL, 17.5 (13.6–23.0) ng/mL, and 39.9 (30.7–53.2) ug/L, respectively. On average, men had significantly higher 25(OH)D and β-CTX concentrations and lower PTH concentrations than women. The concentrations of OC (*p* = 0.307) and P1NP (*p* = 0.961) did not differ significantly between males and females. Age was negatively correlated with the levels of β-CTX (*r* = −0.224; *p*<0.001) and P1NP (*r* = −0.228; *p*<0.001) in men, while it was positively correlated with the levels of β-CTX (*r* = 0.211; *p*<0.001) and OC (*r* = 0.282; *p*<0.001) in women. The levels of 25(OH)D, PTH, and other bone turnover markers were not significantly correlated with age in both genders.

**Table 1:** Clinical and laboratory characteristics.

	Total sample (n = 2588)	Women (n = 1939)	Men (n = 649)	*p* value
Age (years)	43.0±15.7	42.2±15.9	45.5±14.8	<0.001
20–35 years	829 (32.0%)	636 (32.8%)	193 (29.7%)	
36–50 years	793 (30.6%)	586 (30.2%)	207 (31.9%)	
≥51 years	966 (37.3%)	717 (37.0%)	249 (38.4%)	
BMI	22.9±8.6	22.2±9.7	24.8±3.5	<0.001
Albumin (g/L)	50.1±2.6	49.9±2.6	50.6±2.5	<0.001
25(OH)D (ng/mL)	20.9 (16.9–25.0)	20.1 (16.2–24.3)	22.8 (19.1–27.0)	<0.001
PTH (pg/mL)	33.7 (26.3–43.6)	34.4 (27.1–44.0)	31.4 (24.4–41.9)	<0.001
β-CTX (pg/mL)	320.0 (220.0–460.0)	310.0 (210.0–440.0)	370.0 (260.0–512.5)	<0.001
OC (ng/mL)	17.5 (13.6–23.0)	17.5 (13.5–23.2)	17.2 (13.8–22.0)	0.307
P1NP (µg/L)	39.9 (30.7–53.2)	39.9 (30.5–53.6)	39.9 (31.0–52.5)	0.961

Notes: Normally distributed data are shown as mean ± standard. Data that are not normally distributed are shown as medians and inter-quartile ranges (in parentheses).

25(OH)D concentrations were divided into four subgroups according to the following criteria: severely deficient (<10 ng/mL), deficient (≥10 and <20 ng/mL), insufficient (≥20 and <30 ng/mL), and sufficient (≥30 ng/mL). The prevalence of vitamin D insufficiency was 84% in males and 89% in females. The prevalence of vitamin D deficiency was 30% in males and 46% in females. The prevalence of severe vitamin D deficiency was 2% in males and 3.6% in females. The 25(OH)D concentration in men over 60 years was (22.93±7.43) ng/mL, whereas it was (23.91±7.39) ng/mL in those under 60 years. The vitamin D concentration in women over 50 years was (22.70±6.93) ng/mL, whereas it was (20.72±6.91) ng/mL in those under 50 years. There was no significant difference between different age groups for either sex. Because of sex interactions, we analyzed the data separately for males and females. In females (as shown in Table 2), in subgroups with increasing mean 25(OH)D levels, the mean PTH level deceased (by ANOVA, *p*<0.001). In addition, the levels of bone turnover markers, including β-CTX, OC and P1NP, decreased with increasing serum 25(OH)D concentrations (by ANOVA, *p*<0.001). These correlations persisted even after adjustment for age and BMI. In males (as shown in Table 3), in subgroups with increasing mean 25(OH)D concentrations, the mean PTH concentration decreased (by ANOVA, *p*<0.001). In addition, the levels of bone turnover markers, including β-CTX, OC and P1NP, decreased with increasing serum 25(OH)D concentrations (by ANOVA, *p*<0.05). These correlations (except β-CTX: *p* = 0.249 and P1NP: *p* = 0.065) persisted after adjustment for age and BMI.

**Table 2:** Differences in the levels of bone turnover markers in females in different serum 25(OH)D level groups.

Parameter	Serum 25(OH)D concentration (ng/mL)				
	<10	10–20	20–30	>30	*p* value
n	91	852	850	146	
Age (years)	43.1±16.9	41.7±15.8	42.1±16.0	44.6±15.1	0.209
BMI	21.1±2.4	21.9±3.6	22±2.8	22.3±2.6	0.261
Albumin (g/L)	49.9±2.6	50±2.6	49.9±2.5	50±3	0.731
25(OH)D (ng/mL)	7.5 (4.0–8.9)	16.5 (14.2–18.3)	23.4 (21.6–26)	33.3 (31.4–35.7)	<0.001
PTH (pg/mL)	38.7 (30.2–49.9)*** &	35.4 (28.7–45.1)***	33.4 (26.6–43.1) **	29 (21.4–39.1)	<0.001
β-CTX (pg/mL)	350 (240–490)***	325 (222.5–470)***	290 (190–420)	270 (180–435)	<0.001
OC (ng/mL)	18.6 (15.1–28.5)***	18.8 (14.5–24.8)***	16.7 (12.9–21.9)**	15.3 (11.4–20.5)	<0.001
P1NP (µg/L)	42.0 (33.2–63.9)***	41.9 (32.6–55.3)***	38.3 (29.5–51.5)**	34.9 (25.2–46.9)	<0.001

Notes: Normally distributed data are shown as mean ± standard. Data that are not normally distributed are shown as medians and inter-quartile ranges (in parentheses).

*** *p*<0.001 vs. 20–30 ng/mL and >30 ng/mL.

** *p*<0.01 vs. >30 ng/mL.

& *p*<0.05 vs. 10–20 ng/mL.

**Table 3:** Differences in the values of bone turnover markers in males with different levels of serum 25(OH)D.

	Serum 25(OH)D concentration (ng/mL)				
Parameter	<10	10–20	20–30	>30	*p* value
n	19	181	366	83	
Age (years)	41.1±17.2	47.8±16.1	44.4±14	46.3±14.3	0.041
BMI	22.5±2.6	25.2±3.3	24.8±3.7	24.2±2.8	0.077
Albumin (g/L)	50.1±2.9	50.3±2.4	50.7±2.7	50.7±2.1	0.485
25(OH)D (ng/mL)	7.7 (4–8.6)	17.6 (16.1–18.9)	24.2 (22.2–26.6)	34.2 (32.4–39.6)	
PTH (pg/mL)	41.8 (34.9–58.2)*** &	33.5 (25.7–46.1) ***	30.4 (24.3–40.8)	27.4 (21.1–38.2)	<0.001
β-CTX (pg/mL)	470 (320–730)*** &	390 (280–530)	370 (260–510)	325 (232.5–472.5)	0.014
OC (ng/mL)	23 (16.8–26.9)*** &	18.4 (14–24) ***	17 (12.5–21.4)	16 (13.8–19.4)	0.001
P1NP (µg/L)	53 (37.6–67.1)***	42.4 (32.5–54.2) **	39 (30.5–52)	38.3 (29.5–47.5)	0.002

Notes: Normally distributed data are shown as mean ± standard. Data that are not normally distributed are shown as medians and inter-quartile ranges (in parentheses).

*** *p*<0.005 vs. 20–30 ng/mL and >30 ng/mL.

** *p*<0.05 vs. >30 ng/mL.

& *p*<0.05 vs. 10–20 ng/mL.

**Figure 1:**
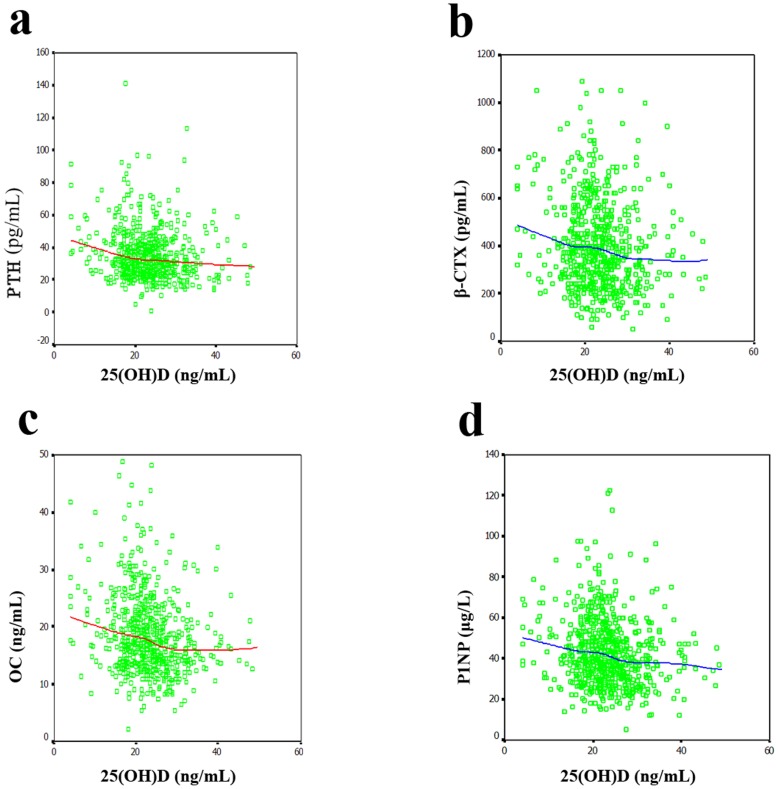
Relationships between the serum 25(OH)D concentration and the concentrations of PTH and bone turnover markers in males. (A) PTH. There was a relatively steep decrease in the concentration of PTH up to 20 ng/mL 25(OH)D and a more gradual decrease between 20 and 40 ng/mL 25(OH)D. This relationship between the serum concentrations of 25(OH)D and PTH exhibited no plateau for serum 25(OH)D levels up to 40 ng/mL. (B–D) β-CTX, OC, and P1NP, respectively. LOESS plots show the plateau levels of bone turnover markers and the 25(OH)D level at which these plateaus are reached, corresponding to 30 ng/mL. When the serum 25(OH)D levels are lower than 30 ng/mL, the levels of the bone turnover markers begin to increase.

**Figure 2:**
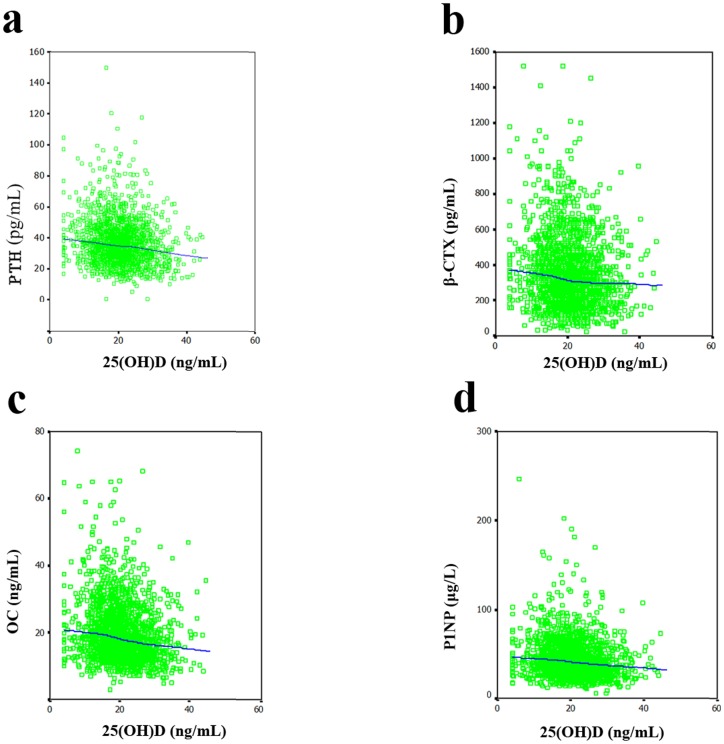
Relationships between serum 25(OH)D concentration and the concentrations of PTH and bone turnover markers in females. (A) PTH. The inverse relationship between the serum concentrations of 25(OH)D and PTH was observed with a gradual decrease up to 40 ng/mL. This relationship between the serum concentrations of 25(OH)D and PTH exhibited no plateau for serum 25(OH)D levels up to 40 ng/mL. (B–D) β-CTX, OC, and P1NP, respectively. LOESS plots show an inverse relationship between the serum concentration of 25(OH)D and bone turnover markers, but no plateau was observed for serum 25(OH)D levels up to 40 ng/mL.

The 25(OH)D level were significantly correlated with the levels of PTH (*r* = −0.139; *p* = 0.035), OC (*r* = −0.188; *p* = 0.004) and P1NP (*r* = −0.146; *p* = 0.025), but not with β-CTX (*r* = −0.083; *p* = 0.206) in males after adjustment for age and BMI. In Fig. 1, the LOESS plots, adjusted for age and BMI, show the mean value of the serum levels of PTH and bone turnover makers for each level of serum 25(OH)D. In the inverse relationship between the serum 25(OH)D and PTH levels (Fig. 1a), there was a relatively steep decrease in PTH up to 20 ng/mL and a relatively slow decrease between 20 and 40 ng/mL. This relationship between the serum 25(OH)D and PTH levels exhibited no plateau with increasing serum 25(OH)D levels up to 40 ng/mL. LOESS plots for the relationships between the serum levels of 25(OH)D and bone turnover markers including β-CTX (Fig. 1b), OC (Figure 1c) and P1NP (Fig. 1d) exhibit plateau concentrations of bone turnover markers; these plateaus occur at a 25(OH)D level of approximately 30 ng/mL. When the serum 25(OH)D concentrations are below 30 ng/mL the levels of bone turnover markers begin to increase. The 25(OH)D level were significantly correlated with the levels of PTH (*r* = −0.188; *p*<0.001), β-CTX (*r* = −0.104; *p* = 0.005), OC (*r* = −0.172; *p*<0.001) and P1NP (*r* = −0.107; *p* = 0.004) in females after adjustment for age and BMI. Fig. 2 shows the correlations of the 25(OH)D levels with the levels of PTH and bone turnover markers in females. An inverse relationship between the serum 25(OH)D and PTH levels (Fig. 2a) was observed, with a gradual decrease up to 40 ng/mL. This relationship between the serum 25(OH)D and PTH levels exhibited no plateau for serum 25(OH)D levels up to 40 ng/mL. In addition, the LOESS plots for the relationships between the levels of 25(OH)D and bone turnover markers including β-CTX (Fig. 2b), OC (Fig. 2c) and P1NP (Fig. 2d) show inverse relationships between the levels of 25(OH)D and those of bone turnover markers, but no plateau was observed for serum 25(OH)D levels up to 40 ng/mL.

## Discussion

Shanghai is located on the eastern coast of China, with a subtropical latitude of 31°N, where vitamin D deficiency was assumed not to be a clinical problem due to the abundant sunshine. However, the results of the present study challenge this assumption. The present study clearly demonstrated that there is a high prevalence of vitamin D insufficiency and/or deficiency among healthy adults living in Shanghai during the winter season. During the winter, the zenith angle is increased, and UVB photons are absorbed by the earth's ozone layer. In addition, this population has a low vitamin D intake because food in China is not supplemented with vitamin D. The results of the present study, together with previous results for Caucasians [12], [17]–[19], Asians [14], [20]–[28], and other men [29], confirm the observation that vitamin D deficiency and/or insufficiency is endemic among populations around the world.

There is still no consensus on the serum 25(OH)D level that defines optimal vitamin D status. However, it is generally agreed that a functional rather than an epidemiological definition should be adopted because serum 25(OH)D concentrations vary with sunshine exposure, season and diet. The proposed criteria to define an optimal level of 25(OH)D include maximum suppression of circulating PTH concentrations, maximum calcium absorption, high bone mineral density, and reduced fracture rates [30], [31]. In agreement with previous studies [27], [32], [33], the present study describes a significant inverse relationship between the serum 25(OH)D and PTH levels independent of age and BMI. This result suggests that vitamin D sufficiency can maintain low serum PTH values. However, although there was a negative correlation between the serum 25(OH)D and PTH levels, there was no threshold values for serum 25(OH)D at which the PTH concentration plateaued at 25(OH)D concentrations less than 40 ng/mL in either males or females. This result was consistent with some studies [23], [27], [28], but not all [12], [34].

The role of vitamin D in the maintenance of bone health is well documented in various populations. However, there is a controversy regarding the relationship between vitamin D and bone turnover markers. Garnero et al. [35] have indicated that vitamin D is unlikely to play a major role in regulating the systemic levels of bone turnover markers. In the present study, as reported in several previous studies [36], [37], we found a significant inverse correlation between the serum levels of 25(OH)D and bone turnover markers, independent of age and BMI. These data suggest that vitamin D insufficiency may be related to accelerated bone turnover, and subsequently, osteoporosis. Several studies [14], [38], [39] have indicated that secondary parathyroidism associated with vitamin D insufficiency is the cause of increased bone resorption, falls and osteoporotic fracture. Allali et al. [40] showed that the correlation between osteocalcin and 25(OH)D is independent of PTH. These findings suggest that vitamin D insufficiency might contribute both directly and indirectly via secondary parathyroidism to accelerated bone turnover. The present study showed that when the serum 25(OH)D concentrations were below 30 ng/mL, the levels of bone turnover markers began to increase in males. The different cutoff points for the serum 25(OH)D level as a marker of the levels of PTH and bone turnover markers seem to paint a biochemical picture in which vitamin D may influence bone turnover directly and indirectly via PTH. When the serum 25(OH)D concentration is below 20 ng/mL, the decrease in the level of serum 25(OH)D will cause a rapid increase in PTH. So, we strongly recommend taking calcium and vitamin D supplements when the serum 25(OH)D concentration is below 20 ng/mL. In addition, for the appropriate maintenance of bone turnover, a level of serum 25(OH)D of 30 ng/mL is suggested. Furthermore, the different cutoff points for serum 25(OH)D as a marker of the levels of PTH and bone turnover markers demonstrate that serum 25(OH)D can influence bone turnover through a new mechanism that is independent of PTH. Therefore, further studies aiming to elucidate the underlying mechanism are needed. Similarly, inverse correlations of serum 25(OH)D with PTH and bone turnover markers were also observed in females (as shown in Fig. 2). However, there were no obvious cutoff points for the serum 25(OH)D concentration associated with appropriate levels of PTH and bone turnover markers. This result might be related to the sex difference and to the fact that serum PTH level is regulated by several other factors including calcium intake.

Secondary hyperparathyroidism results not only from vitamin D deficiency, but also from low calcium intake and low calcium absorption. Similar to other studies [14], [27] conducted in Asian populations, the mean dietary calcium intake of the subjects in the present study was low at (606±232) mg/day. Low dietary calcium intake may contribute to the changes in the serum PTH responses to changes in serum 25(OH)D levels. Therefore, it is important to recommend the use of both vitamin D and calcium supplements.

The strengths of the present study include its large sample size with very strict detailed inclusion criteria. The study population is highly homogeneous, which reduces the effects of potential ethnic and regional confounders that could compromise the estimates. In addition, this study was a population-based study, such that the results can be generalized to all healthy Shanghai adults. The main limitation of the present study was its cross-sectional design; hence, no causal inferences could be made for the observed relations between factors. The participants in this study were sampled from several community centers, and therefore, the selection bias may have been related to the fact that healthy individuals were more likely to participate in the study. In addition, the Roche Elecsys Vitamin D3 (25-OH) immunoassay may overestimate the number of patients with vitamin D deficiency [41].

In conclusion, vitamin D deficiency/insufficiency is very common (30% of men and 46% of women with vitamin D below 20 ng/mL; 84% of men and 89% of women with vitamin D below 30 ng/mL) among healthy adults living in Shanghai. Low serum 25(OH)D levels were associated with both secondary hyperthyroidism and high bone turnover status. Further studies aiming to elucidate the underlying mechanism by which serum 25(OH)D affects bone turnover are needed.

## References

[1] Pittas AG, Lau J, Hu FB, Dawson-Hughes B (2007) The role of vitamin D and calcium in type 2 diabetes. A systematic review and meta-analysis. J Clin Endocrinol Metab 92: 2017–2029. 17389701 10.1210/jc.2007-0298 PMC2085234

[2] Svoren BM, Volkening LK, Wood JR, Laffel LM (2009) Significant vitamin D deficiency in youth with type 1 diabetes mellitus. J Pediatr 154: 132–134. 19187735 10.1016/j.jpeds.2008.07.015 PMC2635941

[3] Garland CF, Gorham ED, Mohr SB, Garland FC (2009) Vitamin D for cancer prevention: global perspective. Ann Epidemiol 19: 468–483. 19523595 10.1016/j.annepidem.2009.03.021

[4] Munger KL, Zhang SM, O'Reilly E, Hernan MA, Olek MJ, et al (2004) Vitamin D intake and incidence of multiple sclerosis. Neurology 62: 60–65. 14718698 10.1212/01.wnl.0000101723.79681.38

[5] Cutolo M, Otsa K (2008) Review: vitamin D, immunity and lupus. Lupus 17: 6–10. 18089676 10.1177/0961203307085879

[6] Holick MF (2010) Vitamin D: extraskeletal health. Endocrinol Metab Clin North Am 39: 381–400. 20511059 10.1016/j.ecl.2010.02.016

[7] Wang TJ, Pencina MJ, Booth SL, Jacques PF, Ingelsson E, et al (2008) Vitamin D deficiency and risk of cardiovascular disease. Circulation 117: 503–511. 18180395 10.1161/CIRCULATIONAHA.107.706127 PMC2726624

[8] Cannell JJ, Vieth R, Umhau JC, Holick MF, Grant WB, et al (2006) Epidemic influenza and vitamin D. Epidemiol Infect 134: 1129–1140. 16959053 10.1017/S0950268806007175 PMC2870528

[9] Webb AR (2006) Who, what, where and when-influences on cutaneous vitamin D synthesis. Prog Biophys Mol Biol 92: 17–25. 16766240 10.1016/j.pbiomolbio.2006.02.004

[10] Holick MF, Chen TC (2008) Vitamin D deficiency: a worldwide problem with health consequences. Am J Clin Nutr 87: 1080S–1086S. 18400738 10.1093/ajcn/87.4.1080S

[11] Lips P (2004) Which circulating level of 25-hydroxyvitamin D is appropriate? J Steroid Biochem Mol Biol 89–90: 611–614. 10.1016/j.jsbmb.2004.03.040 15225848

[12] Chapuy MC, Preziosi P, Maamer M, Arnaud S, Galan P, et al (1997) Prevalence of vitamin D insufficiency in an adult normal population. Osteoporos Int 7: 439–443. 9425501 10.1007/s001980050030

[13] Holick MF (2007) Vitamin D deficiency. N Engl J Med 357: 266–281. 17634462 10.1056/NEJMra070553

[14] Holick MF (2008) The vitamin D deficiency pandemic and consequences for nonskeletal health: mechanisms of action. Mol Aspects Med 29: 361–368. 18801384 10.1016/j.mam.2008.08.008 PMC2629072

[15] Wat WZ, Leung JY, Tam S, Kung AW (2007) Prevalence and impact of vitamin D insufficiency in southern Chinese adults. Ann Nutr Metab 51: 59–64. 17356256 10.1159/000100822

[16] Zhao J, Xia W, Nie M, Zheng X, Wang Q, et al (2011) The levels of bone turnover markers in Chinese postmenopausal women: Peking Vertebral Fracture study. Menopause 18: 1237–1243. 21747303 10.1097/gme.0b013e31821d7ff7

[17] Lips P, Duong T, Oleksik A, Black D, Cummings S, et al (2001) A global study of vitamin D status and parathyroid function in postmenopausal women with osteoporosis: baseline data from the multiple outcomes of raloxifene evaluation clinical trial. J Clin Endocrinol Metab 86: 1212–1221. 11238511 10.1210/jcem.86.3.7327

[18] Bettica P, Bevilacqua M, Vago T, Norbiato G (1999) High prevalence of hypovitaminosis D among free-living postmenopausal women referred to an osteoporosis outpatient clinic in northern Italy for initial screening. Osteoporos Int 9: 226–229. 10450411 10.1007/s001980050141

[19] van Dam RM, Snijder MB, Dekker JM, Stehouwer CD, Bouter LM, et al (2007) Potentially modifiable determinants of vitamin D status in an older population in the Netherlands: the Hoorn Study. Am J Clin Nutr 85: 755–761. 17344497 10.1093/ajcn/85.3.755

[20] Goswami R, Gupta N, Goswami D, Marwaha RK, Tandon N, et al (2000) Prevalence and significance of low 25-hydroxyvitamin D concentrations in healthy subjects in Delhi. Am J Clin Nutr 72: 472–475. 10919943 10.1093/ajcn/72.2.472

[21] Arya V, Bhambri R, Godbole MM, Mithal A (2004) Vitamin D status and its relationship with bone mineral density in healthy Asian Indians. Osteoporos Int 15: 56–61. 13680103 10.1007/s00198-003-1491-3

[22] Vupputuri MR, Goswami R, Gupta N, Ray D, Tandon N, et al (2006) Prevalence and functional significance of 25-hydroxyvitamin D deficiency and vitamin D receptor gene polymorphisms in Asian Indians. Am J Clin Nutr 83: 1411–1419. 16762954 10.1093/ajcn/83.6.1411

[23] Harinarayan CV, Ramalakshmi T, Prasad UV, Sudhakar D, Srinivasarao PV, et al (2007) High prevalence of low dietary calcium, high phytate consumption, and vitamin D deficiency in healthy south Indians. Am J Clin Nutr 85: 1062–1067. 17413106 10.1093/ajcn/85.4.1062

[24] Yan L, Prentice A, Zhang H, Wang X, Stirling DM, et al (2000) Vitamin D status and parathyroid hormone concentrations in Chinese women and men from north-east of the People's Republic of China. Eur J Clin Nutr 54: 68–72. 10694775 10.1038/sj.ejcn.1600895

[25] Nakamura K, Nashimoto M, Matsuyama S, Yamamoto M (2001) Low serum concentrations of 25-hydroxyvitamin D in young adult Japanese women: a cross sectional study. Nutrition 17: 921–925. 11744341 10.1016/s0899-9007(01)00662-1

[26] Ono Y, Suzuki A, Kotake M, Zhang X, Nishiwaki-Yasuda K, et al (2005) Seasonal changes of serum 25-hydroxyvitamin D and intact parathyroid hormone levels in a normal Japanese population. J Bone Miner Metab 23: 147–151. 15750693 10.1007/s00774-004-0553-8

[27] Ardawi MS, Sibiany AM, Bakhsh TM, Qari MH, Maimani AA (2012) High prevalence of vitamin D deficiency among healthy Saudi Arabian men: relationship to bone mineral density, parathyroid hormone, bone turnover markers, and lifestyle factors. Osteoporos Int 23: 675–686. 21625888 10.1007/s00198-011-1606-1

[28] Ho-Pham LT, Nguyen ND, Lai TQ, Eisman JA, Nguyen TV (2011) Vitamin D status and parathyroid hormone in a urban population in Vietnam. Osteoporos Int 22: 241–8. 20414642 10.1007/s00198-010-1207-4

[29] Meddeb N, Sahli H, Chahed M, Abdelmoula J, Feki M, et al (2005) Vitamin D deficiency in Tunisia. Osteoporos Int 16: 180–183. 15197539 10.1007/s00198-004-1658-6

[30] Heaney RP (1999) Lessons for nutritional science from vitamin D. Am J Clin Nutr 69: 825–826. 10232617 10.1093/ajcn/69.5.825

[31] McKenna MJ, Freaney R (1998) Secondary hyperparathyroidism in the elderly: means to defining hypovitaminosis D. Osteoporos Int 8 Suppl 2 3S–6S. 10.1007/pl00022725 10197175

[32] Souberbielle JC, Cormier C, Kindermans C, Gao P, Cantor T, et al (2001) Vitamin D status and redefining serum parathyroid hormone reference range in the elderly. J Clin Endocrinol Metab 86: 3086–3090. 11443171 10.1210/jcem.86.7.7689

[33] Pepe J, Romagnoli E, Nofroni I, Pacitti MT, De Geronimo S, et al (2005) Vitamin D status as the major factor determining the circulating levels of parathyroid hormone: a study in normal subjects. Osteoporos Int 16: 805–812. 15551058 10.1007/s00198-004-1757-4

[34] Saliba W, Barnett O, Rennert HS, Lavi I, Rennert G (2011) The relationship between serum 25(OH)D and parathyroid hormone levels. Am J Med 124: 1165–1170. 22114830 10.1016/j.amjmed.2011.07.009

[35] Garnero P, Munoz F, Sornay-Rendu E, Delmas PD (2007) Associations of vitamin D status with bone mineral density, bone turnover, bone loss and fracture risk in healthy postmenopausal women. The OFELY study. Bone 40: 716–722. 17112798 10.1016/j.bone.2006.09.026

[36] Gannage-Yared MH, Chemali R, Yaacoub N, Halaby G (2000) Hypovitaminosis D in a sunny country: relation to lifestyle and bone markers. J Bone Miner Res 15: 1856–1862. 10977006 10.1359/jbmr.2000.15.9.1856

[37] Diamond TH, Levy S, Smith A, Day P (2002) High bone turnover in Muslim women with vitamin D deficiency. Med J Aust 177: 139–141. 12149082 10.5694/j.1326-5377.2002.tb04699.x

[38] Visser M, Deeg DJ, Lips P (2003) Low vitamin D and high parathyroid hormone levels as determinants of loss of muscle strength and muscle mass (sarcopenia): the Longitudinal Aging Study Amsterdam. J Clin Endocrinol Metab 88: 5766–5772. 14671166 10.1210/jc.2003-030604

[39] Sambrook PN, Chen JS, March LM, Cameron ID, Cumming RG, et al (2004) Serum parathyroid hormone predicts time to fall independent of vitamin D status in a frail elderly population. J Clin Endocrinol Metab 89: 1572–1576. 15070914 10.1210/jc.2003-031782

[40] Allali F, El Aichaoui S, Khazani H, Benyahia B, Saoud B, et al (2009) High prevalence of hypovitaminosis D in Morocco: relationship to lifestyle, physical performance, bone markers, and bone mineral density. Semin Arthritis Rheum 38: 444–451. 18336870 10.1016/j.semarthrit.2008.01.009

[41] Connell AB, Jenkins N, Black M, Pasco JA, Kotowicz MA, et al (2011) Overreporting of vitamin D deficiency with the Roche Elecsys Vitamin D3 (25-OH) method. Pathology 43: 368–371. 21566493 10.1097/PAT.0b013e328346431c

